# SARS-CoV-2 and COVID-19: A perspective from environmental virology

**DOI:** 10.1590/1678-4685-GMB-2020-0228

**Published:** 2021-03-08

**Authors:** Meriane Demoliner, Juliana Schons Gularte, Viviane Girardi, Paula Rodrigues de Almeida, Matheus Nunes Weber, Ana Karolina Antunes Eisen, Juliane Deise Fleck, Fernando Rosado Spilki

**Affiliations:** 1 Universidade Feevale, Laboratório de Microbiologia Molecular, Novo Hamburgo, RS, Brazil.; 2 Universidade Feevale, Programa de Pós-Graduação em Qualidade Ambiental, Novo Hamburgo, RS, Brazil.; 3 Universidade Feevale, Mestrado Acadêmico em Virologia, Novo Hamburgo, RS, Brazil.

**Keywords:** SARS-CoV-2, COVID-19, environmental virology, Brazil, disease pandemic

## Abstract

December 2019 marked the beginning of the current Coronavirus disease pandemic (COVID-19). *Severe acute respiratory syndrome-related coronavirus 2* (SARS-CoV-2) was identified as the causative agent of a viral pneumonia outbreak in Wuhan, Hubei Province, China. The alarming spread levels and clinical severity elevated the status of COVID-19 to the global pandemic by the World Health Organization. In 6 months, more than 25 million cases of infected people and more than 890,000 deaths by COVID-19 had been reported worldwide. The main goal of this review is to shed light upon the current COVID-19 epidemic situation in Brazil with a health approach highlighting some unique environmental, animal and epidemiological aspects.

## Introduction

*Severe acute respiratory syndrome-related coronavirus 2* (SARS-CoV-2), initially named Novel coronavirus (2019-nCoV), is the causative agent of the current Coronavirus disease 2019 (COVID-19) pandemic (Gorbalenya *et al.,* 2020). SARS-CoV-2 belongs to the *Coronaviridae* family (subfamily *Coronavirinae*) which includes four genera, *Alpha-, Beta-, Gamma-* and *Delta- coronavirus*. Only alpha and betacoronaviruses are known to infect humans, of which human coronavirus (HCoV)-229E and HCoV-NL63 are classified as alphacoronaviruses, while HCoV-HKU1, HCoV-OC43, *Middle East respiratory syndrome coronavirus* (MERS-CoV), SARS-CoV, and SARS-CoV-2 are grouped within the *Betacoronavirus* genus.

The subgenus *Sarbecovirus* comprises SARS-CoV and SARS-CoV-2, which are classified in the same species: SARS-related coronavirus (SARSr-CoV) (Cui *et al*., 2019). This viral family is characterized by significant genetic variability and a high recombination rate that enable them to be easily distributed among humans and animals worldwide. Coronaviruses are enveloped and have a positive-sense single-stranded RNA genome of approximately 30 kb, ranging from 60-220 nm in size with crown-like spike proteins on their surfaces that bind to host cell proteins allowing viral entry (Tu *et al*., 2020).

In December 2019, this virus caused viral pneumonia in Wuhan, Hubei Province, China, being identified in the first week of 2020 with fast dissemination throughout China and surrounding countries (Jin *et al*., 2020). The alarming levels and severity of its spreading elevated the status of COVID-19 to the global pandemic by the World Health Organization (WHO) on March 11^th^, 2020. On that date, the number of cases outside China had increased 13-fold and the number of affected countries had tripled (more than 118,000 cases in 114 countries, 4,291 deaths) (WHO, 2020a).

The epidemiological situation worldwide has been changing since the beginning of the outbreak. According to WHO (2020b), the Western Pacific region had the majority of the daily confirmed cases in the first months. After that, the situation had rapidly changed and the highest daily confirmed cases occurred in the Eastern Mediterranean region at the beginning of March, followed by Europe from March to April, alternating some days with the Americas, and since April 21^st^ the Americas have been showing the highest numbers so far (data updated on September 9^th^, 2020). By the beginning of September of 2020, more than 25 million cases and more than 890,000 deaths due COVID-19 have been reported worldwide (WHO, 2020b, c).

In Europe, the first patient was diagnosed with COVID-19 on January 24^th^, 2020, a 48-year-old French woman who came from China (Lescure *et al*., 2020). On September 2020, the country in Europe with the highest number of infected people was the Russian Federation, with more than 1 million confirmed cases (WHO, 2020b).

In the Americas region, the United States of America (USA) and Brazil have the highest numbers of confirmed cases and deaths from COVID-19 (WHO, 2020b, c). In the USA, the first case of COVID-19 was reported on January 19^th^, 2020, a 35-year-old man in the Snohomish County, Washington, with a four-day history of cough and fever. He revealed that he had returned to Washington State on January 15^th^, after traveling to visit his family in Wuhan, China (Holshue *et al*., 2020). In September 2020, the total number of confirmed cases in the USA was more than 6 million (WHO, 2020b).

In Latin America, the first case of COVID-19 was confirmed in Brazil by the Ministry of Health on February 25^th^, 2020. The case was a Brazilian man, 61 years-old, who traveled from February 9^th^ to 20^th^, 2020, to Lombardy, Northern Italy (Rodriguez-Morales *et al*., 2020). In September 2020, Brazil already had more than 4 million confirmed cases and more than 100 thousand deaths (WHO, 2020b). The current data showed that four out of ten countries with the highest number of infections were from South America (Brazil, Peru, Colombia and Argentina) (WHO, 2020c). Globally, in September 2020, the Americas had the highest number of infected people, more than 14 million cases; while the Western Pacific had the lowest number with about 500 thousand cases. In relation to the highest number of deaths, the Americas recorded the highest number, almost 500 thousand deaths, and Western Pacific had the lowest with about 11 thousand deaths (WHO, 2020b, c).

The main goal of this review is to shed light upon the current COVID-19 epidemic situation in Brazil with a health approach, highlighting some unique environmental, animal and epidemiological peculiarities that impose challenges to overcome this situation in a society with high levels of inequality. Environmental factors are associated with the spillover of SARS-CoV-2 and might constitute the main driving source of the persistence of the pandemics.

## The spread of SARS-CoV-2 in Brazil

São Paulo-Guarulhos International Airport, located in the State of São Paulo, is the largest airport in Brazil and this city is the most populated in South America (> 23 million people). Furthermore, besides the high air traffic connecting Brazil with cities in Latin America, North America, Europe, Africa and the Middle East, there are usual connections with metropolitan centers of Paraguay, Argentina, Uruguay and Bolivia by roads and bus services, and with Chile, Argentina and Bolivia by some railways (Rodriguez-Morales *et al*., 2020).

So, without border control, the spread of SARS-CoV-2 through these routes is probably inevitable. According to Candido *et al*. (2020), it is estimated that 54.8% of SARS-CoV-2 introductions in Brazil came from travelers infected in Italy, 9.3% in China and 8.3% in France. Only the route Italy - São Paulo could be responsible to comprise 24.9% of the total of travelers with COVID-19 flying to Brazil during this period.

COVID-19 was declared a pandemic on March 11^th^, 2020, but only on March 27^th^, the Brazilian government issued a new ordinance (No. 152) restricting the entry of foreigners of all nationalities to Brazil (Governo Federal do Brasil, 2020). On the same day the Centers for Disease Control and Prevention (CDC) issued a Global COVID-19 Level 3 Warning recommending that travelers avoided all nonessential international travels (CDC, 2020a).

As of March 30^th^, 2020, there was no official protocol for SARS-CoV-2 testing in ports, airports and borders; the only measure adopted by the National Health Surveillance Agency (Agência Nacional de Vigilância Sanitária - ANVISA) was to identify symptomatic cases of COVID-19 at such locations (ANVISA, 2020). However, only symptom screening of incoming passengers may not be effective to reduce the global spread of SARS-CoV-2, as a large proportion of infected travelers may be asymptomatic or presymptomatic. That way, to decrease the risk of importation of cases to other countries it is necessary to improve the tracing within the epicenters (Wells *et al*., 2020).

As described above, SARS-CoV-2 was first reported in Brazil from a traveler coming from Italy. At the beginning of SARS-CoV-2 spread in Brazil, most of the diagnosis was performed in higher-income people (Souza *et al*., 2020). People with that social condition, for example, traveled more during summer vacation and many were infected abroad. Thus, the infection to low-income people that comprises most of the Brazilian population occurred later. Another Brazilian study showed that less-educated adults presented a 2-fold higher prevalence of SARS-CoV-2 infections (Rezende *et al*., 2020).

A few weeks after the first detection in Brazil, local transmission was already established in several foci (Souza *et al*., 2020), and once the virus reached the low-income classes, control measures became more difficult to be taken since several official diagnostic laboratories became initially overwhelmed. Currently, the lack of personnel and laboratory material to meet the demand for diagnosis in official government laboratories has been met by hospitals and research groups from universities in several Brazilian states.

## Regional climate and SARS-CoV-2 transmission

In the past, a study showed that high temperatures and high relative humidity have a negative effect on SARS-CoV viability, while lower temperatures and low humidity can be a benefit to prolong virus survival on contaminated surfaces (Chan *et al*., 2011). In the beginning, the first studies with SARS-CoV-2 showed similar stability to that of SARS-CoV, for which the main results demonstrated that at 65% relative humidity and 21-23 °C temperature the virus can be viable in the air for up 3 hours (Van Doremalen *et al*., 2020).

When the stability of SARS‐CoV‐2 is evaluated at different temperatures, it is possible to observe that this virus is highly stable at 4 °C and sensitive to the heat. As the temperature rises, the time that the virus remains viable decreases, for example, at 22 °C the virus can remain viable for 7 days, at 37 °C for 1 day, at 56 °C for 10 minutes and at 70 °C for 1 minute (Chin *et al*., 2020).

Brazil is a large tropical country with most of its territory located between the Equator and the Tropic of Capricorn. A Brazilian study about the effect of tropical weather on SARS-CoV-2 transmission did not identify evidence of a negative correlation on COVID-19 infection in higher temperatures (above 25 °C). However, below the threshold of 25.8 °C, the cases decreased by −5.9035 for every 1 °C rise in temperature (Prata *et al*., 2020).

The highest annual temperature means are usually in the North and the lowest in the South, however, the South of Brazil is the region that has been presenting the lowest number of cases and deaths per million inhabitants so far (data updated on August 15^th^, 2020) (Table 1) and showed an increase in case numbers later when compared to other states.

On the other hand, in the first months of the pandemic, the Northeast and North regions have quickly shown a spread in cases and death numbers, being only behind the Southeast region. The North region has also shown the highest number of deaths per million inhabitants (Table 1). These data show us that questions regarding the regional differences of a country as large and diverse as Brazil may have a more relevant influence on the spread and aggravation of the disease when compared to abiotic issues.

**Table 1 -:** Number of cases and deaths by COVID-19 in different regions of Brazil*.

	Cases of COVID-19		Deaths by COVID-19	
Region of Brazil	Cumulative number	Per million inhabitants	Cumulative number	Per million inhabitants
Southeast	1,158,423	1,310.90	48,214	54.6
Northeast	1,018,476	1,784.60	32,108	56.30
North	473,725	2,570.30	12,670	68.7
South	321.217	1,071,60	7,079	23.6
Center-West	345,255	2,188.50	7,161	43.9

*The data of cases and deaths of COVID-19 in the regions of Brazil for the 33rd epidemiological week was obtained from the 27th Special Epidemiological Bulletin COE-COVID-19 published by the Ministry of Health and the Health Surveillance Secretariat (SVS) of Brazil (Ministério da Saúde do Brasil, 2020).

## Social distancing

To reduce SARS-CoV-2 transmission, an early implementation of social distancing has proven effective in many countries. Also, it is much important to accelerate and expand testing to obtain data more accurately in order to be able to track positive cases and contact people who had been in contact with those cases to better control COVID-19 spread (Bedford *et al*., 2020; de Oliveira *et al*., 2020). Since there are no COVID-19 vaccines widely available so far, non-pharmacological interventions like social distancing, quarantine and isolation of infected individuals are important alternatives to reduce transmission and slow the SARS-CoV-2 spread (Wilder-Smith and Freedman, 2020). Therefore, flexible social distancing measures must be made with extreme caution.

The Brazilian Inloco startup developed a map showing the percentage of the population that is respecting the isolation recommendation during the COVID-19 pandemic based on the location of their cell phones, and although it is not possible to represent the total population it is possible to have an approximate number of the Social Isolation Index. The data demonstrated that the higher index achieved on May 22^nd^, 2020, where the Social Isolation Index was 62.6%, this number has been falling, and on June 26^th^, 2020, the index was 37.5% (Inloco, 2020).

On May 8^th^, 2020, in its Report 21, the Imperial College COVID-19 Response Team published that in Brazil, so far, the changes in mobility have not been stringent enough to reduce the transmission number and it is possible to project a continued growth of the epidemic. Therefore, if the government does not adopt harder control measures the virus will still have enough hosts to infect, the epidemic will continue to grow exponentially and the associated number of cases and deaths will therefore increase (Mellan *et al*., 2020).

## Initiatives of Brazilian research groups to help in the SARS-CoV-2 pandemic

In Brazil there are State Central Laboratories (*Laboratórios Centrais de Saúde Pública* - LACEN) that belong to the National System of Public Health Laboratories (Sistema Nacional de Laboratórios de Saúde Pública - SISLAB). Currently, they do the most of the diagnosis of SARS-CoV-2. However, only these institutions and private diagnosis labs are not enough to cover the high diagnosis demand imposed by the COVID-19 pandemic.

Therefore, to contribute to a faster and efficient national response to the present pandemic, many research groups offered their labs and expertise to accelerate the diagnosis. The tests done in the laboratories from public and private universities counted mainly with the help from researchers and student volunteers. However, even with these efforts the number of COVID-19 tests performed is still low.

According to the Worldometer Website (September 2020), Brazil occupies the rank position in the number of tests performed and 10^th^ in the number of confirmed cases per 1 million people. Mass testing is seen as an important method to identify the positive cases and apply public health measures in time. These actions help to slow transmission and develop strategies to decrease the impacts of the pandemic on society.

In addition to the huge help in the diagnosis, many Brazilian scientists are working in several research projects about SARS-CoV-2 and COVID-19, including the development of diagnostic kits, testing of drugs and vaccines, and on the production of personal protective equipment and respirators.

## SARS-CoV-2 in animals

Coronaviruses are known to infect wild and domestic animals, where bats act as a reservoir for different CoV genera and species (Banerjee *et al*., 2019). Alpha and beta-CoVs are frequently reported in mammals, while delta and gamma-CoV are more frequently reported in birds. SARS-CoV-1 was the first CoV known to have inflicted severe disease in humans. It is likely that SARS-CoV-1 has an evolutionary origin in horseshoe bats (*Rhinolophus* spp.) (Wong *et al*., 2019), but as this virus was also found in captive cat civets (*Civettictis civetta*) and raccoon dogs (*Nyctereutes procyonoides*) in markets and some farms, it is believed that these species might have acted as intermediate hosts for SARS-CoV-1 (Guan *et al*., 2003; Kan *et al*., 2005).

SARS-CoV-2 is phylogenetically closely related to SARS-CoV-1, and it has been classified in the same betacoronavirus species; besides, early genomic comparisons revealed that the most closely related viruses to SARS-CoV-2 came from bats (Zhou *et al*., 2020). Although bats are likely to be the reservoir and host for this virus, their general ecological separation from humans makes it probable that other mammalian species act as intermediate or amplifying hosts, within which SARS-CoV-2 was able to acquire some or all of the mutations needed for efficient human transmission (Zhang and Holmes, 2020). According to Zang and Holmes (2020), to determine what these intermediate host species might be, it is imperative to perform a much wider sampling of animals from wet markets or that live close to human populations. Moreover, other SARS-CoVs were reported in different bat species worldwide (Góes *et al*., 2013).

SARS-CoV-2 spread in Brazil initially affecting the Southeastern, Northeastern and Northern regions. The Southeastern region has high demographic density, whereas the other two regions present poorer sanitation and public healthcare conditions. Of great concern is the spreading to the Amazon region, which combined with alarming levels of deforestation, poses an enormous threat to native indigenous tribes and creates conditions for this virus to come in contact with wildlife (Ellwanger *et al*., 2020). Brazil presents around 184 bat species, and such species richness is similar to that found in the Southeast of Asia; albeit adaptation to a novel host and a zoonotic establishment is a complex multifactorial process, this fact should be considered when predicting the risk of the perpetuation of SARS-CoV-2 in South America (Han *et al*., 2016).

This scenario is considered in North America (Franklin and Bevins, 2020) where bat diversity, contact with humans and richness are far lower than in Brazil, therefore this form of perpetuation should be monitored in the future in Brazil as well. Currently, few studues are searching for CoV in Brazilian bats, where only alpha-CoV was found to date (Góes *et al*., 2013). The wide variety of beta-CoV found in bats worldwide combined with the heterogeneity of Brazilian bats may be a potential source of CoV investigation.

Recent research shows the presence of viruses closely related to SARS-CoV-2 in Malayan pangolins (*Manis javanica*), that are of great interest since they are frequently involved in illegal trafficking and their endangered status. Moreover, the recent studies that showed that they can carry viruses closely-related to SARS-CoV-2 suggest a far greater diversity of related betacoronaviruses in a variety of mammalian species. However, to fully understand the extent of hosts and the diversity of betacoronaviruses circulating, animal samples need to be further investigated. On the other hand, some authors report that the potential amplifying mammalian host, intermediate between bats and humans, is unknown.

Since the mutation in the original strain could directly have triggered virulence towards humans, it is not certain that this intermediary exists (Cascella *et al*., 2020). Many uncertainties due to lack of data remain for the actual hosts of SARS-CoV-2 and their zoonotic characteristics, showing the importance of including monitoring and surveillance of this virus in wild animals. As a way to minimize these uncertainties, (Becker *et al*. (2020) carried out a study proposing predictions models that could help to guide sampling for novel potentially zoonotic viruses, immunological research to characterize key receptors (e.g., ACE2) and identify mechanisms of viral tolerance, and experimental infections to quantify competence of suspected host species.

It has been experimentally shown that among animals, felids and mustelids are permissive to SARS-CoV-2 infection, while dogs, pigs, chickens and ducks are not as susceptible (Shi *et al.*, 2020). A recent outbreak of SARS-CoV-2 in mink farms in the Netherlands raised serious concerns about the susceptibility of this taxon to SARS-CoV-2 disease (Oreshkova *et al*., 2020). Differently to what was reported in felids, in which only mild disease in a low number of animals was observed, the outbreak in Dutch minks presented cases of severe pneumonia, mortality and a high number of infected animals.

This example raises concerns about possible spillover between humans and these mustelids, since there are six genera from the Mustelidae family in South America and more than a dozen species of felids. With the current anthropogenic disturbance of the habitats of these animals in Brazil, there is a risk of infection of endangered species and spillover events that could perpetuate SARS-CoV-2 in nature and allow new mutations and possible new viral variants to occur as the virus interacts with new hosts and is challenged with a different immunological response (Ellwanger *et al*., 2020). Moreover, the high pressure of infection during the SARS-CoV-2 pandemic may allow a putative permissive new host adaptation.

## SARS-CoV-2 in aquatic environments

Human enteric viruses (which are non-enveloped viruses) are known as the main pathogens that cause water-borne diseases. Among the diseases, we could mention gastrointestinal, conjunctivitis, respiratory symptoms and viral hepatitis. On the other hand, enveloped viruses behave differently in the aquatic environment. In general, these viruses are not considered as a major threat to the wastewater and water industries due to their supposedly low concentration and high susceptibility to degradation in the aquatic environment. However, several clinical reports show that certain enveloped viruses are excreted in the faeces during infection (Wigginton *et al*., 2015).

The transmission mode of SARS-CoV-2 occurs through person-to-person contact through respiratory droplets, sneezing, coughing, direct contact with an infected individual, or indirect contact through fomites. Water transmission has never been demonstrated in humans, but since SARS-CoV-2 was found in stool samples (Holshue *et al*., 2020; Xiao *et al*., 2020) the possibility of fecal-oral transmission needs to be clarified. La Rosa *et al*. (2020a) carried out a review work to investigate publications on CoV in aquatic environments. The authors mainly reported that: CoV appears to be inactivated faster in water than non-enveloped human enteric viruses; temperature is an important factor influencing viral survival (the infectious viral titer decreases more quickly at 23 °C and 25 °C than at 4 °C), and so far, there is no evidence that CoV is present in surface or groundwater or that it is transmitted through contaminated drinking water.

According to the Centers for Disease Control and Prevention (CDC), so far, SARS-CoV-2 has not been detected in drinking water, since conventional water treatment methods that use filtration and disinfection used in public drinking water treatments are efficient on virus removal or inactivation. Also, the CDC states that there is no evidence that the virus can be transmitted to people through the water in swimming pools, hot tubs or water playgrounds.

This is possible as long as adequate water disinfection is performed, thus ensuring viral inactivation. It is worth mentioning that everyone should follow state, local, territorial or tribal guidance that might determine when and how public pools, hot tubs or water playgrounds may operate and might include CDC considerations (CDC, 2020b).

In addition to the respiratory symptoms caused by COVID-19, a varied percentage of patients (between 16% and 73%) have reported diarrhea as an additional symptom. Furthermore, recent studies show the presence of SARS-CoV-2 genetic material in patients' stool samples (Holshue *et al*., 2020; Xiao *et al*., 2020; Xu *et al*., 2020). Several studies in many countries, such as Netherlands (Medema *et al*., 2020), United States (Wu *et al*., 2020), Australia (Ahmed *et al*., 2020), France (Wurtzer *et al*., 2020) and Italy (La Rosa *et al*., 2020b) have demonstrated the occurrence of SARS-CoV-2 in municipal wastewater.

Although no studies are showing SARS-CoV-2 transmission by wastewater, the increase in the viral circulation in the population overloads municipal sewage systems. Thus, it is worth highlighting the importance of viral monitoring in these environments, so that it is possible to obtain information on the risk for workers from the wastewater treatment plant and also to monitor the circulation of SARS-CoV-2 in the community, thus complementing clinical surveillance (Medema *et al*., 2020).

In Italy, La Rosa *et al*. (2020b) evaluated the presence of SARS-CoV-2 RNA in 12 sewage samples collected between February and April 2020 at the wastewater treatment plants in Milan and Rome of high and low endemic circulation areas, respectively. Overall, 6 of the 12 samples were positive, one of which was obtained from Milan wastewater collected a few days after the first Italian case of COVID-19. Medema *et al*. (2020) conducted a study on sewage samples in seven cities in the Netherlands using the RT-PCR method. No positive SARS-CoV-2 samples were detected on February 6^th^, three weeks before the first case was reported in the Netherlands, on February 27^th^. On the other hand, in general, between March 5^th^ and 16^th^, positive samples were detected in six sites.

In France, Wurtzer *et al*. (2020) carried out a study on samples collected from the wastewater treatment plant of the Parisian area from March 5^th^ to April 23^rd^, 2020 (including the lockdown period since March 17^th^, 2020). They reported that the increase of genome units in raw wastewaters accurately followed the increase of human COVID-19 cases observed at the regional level and that the viral genomes fragments (E gene) could be detected before the beginning of the exponential growth of the epidemic. The authors also claim that a marked decrease in the quantities of genomes units was observed concomitantly with the reduction in the number of new COVID-19 cases as an expected consequence of the lockdown.

Many cities in Brazil do not receive proper sanitation infrastructures nor have proper sewage treatment before it is disposed of back into the watersheds, and several infectious agents have been detected in water, which is a source of microbial source tracking in different environments (Spilki *et al*., 2013). Hence, SARS-CoV-2 detection in wastewater and in watersheds may occur in Brazil, offering another source to assess viral spread.

## Conclusion

Brazil had around two months to prepare strategies to face the COVID-19 pandemic, and considering the time and the other countries' examples that were affected first, it could have had better results, which it did not happen. Brazil is a developing country with a large social inequality and now, besides a sanitary crisis, it is also facing a political crisis. During the pandemic, two Health Ministers were withdrawn from their position, and until now (June 26th, 2020) no other Minister was appointed yet. As the country presents high numbers of cases and deaths by COVID-19, it is still well known that there is a large number of unreported cases.

Another problem is that while the number of positive cases of COVID-19 increased, there was a decrease in social distancing, an unusual event. Moreover, Brazil presents a plethora of putative wild animal candidates to harbor a possible spillover, including mainly bats, rodents, and mustelids. The anthropogenic disturbance of wildlife habitats added to the environmental contamination may be a potential risk factor that must not be ignored. Reducing deforestation combined with continuous evaluation of the betacoronaviruses circulation on these species may be a potential prevention measure to avoid new outbreaks with a zoonotic origin.

## References

[B1] Ahmed W, Angel N, Edson J, Bibby K, Bivins A, O’Brien JW, Choi PM, Kitajima M, Simpson SL, Li S (2020). First confirmed detection of SARS-CoV-2 in untreated wastewater in Australia: A proof of concept for the wastewater surveillance of COVID-19 in the community. Sci Total Environ.

[B2] Banerjee A, Kulcsar K, Misra V, Frieman M, Mossman K (2019). Bats and coronaviruses. Viruses.

[B3] Becker DJ, Albery GF, Sjodin AR, Poisot T, Dallas TA, Eskew EA, Farrell MJ, Guth S, Han BA, Simmons NB (2020). Predicting wildlife hosts of betacoronaviruses for SARS-CoV-2 sampling prioritization. bioRxiv.

[B4] Bedford J, Enria D, Giesecke J, Heymann DL, Ihekweazu C, Kobinger G, Lane HC, Memish Z, Oh M, Sall AA (2020). COVID-19: towards controlling of a pandemic. Lancet.

[B5] Candido DS, Watts A, Abade L, Kraemer MUG, Pybus OG, Croda J, Oliveira W, Khan K, Sabino EC, Faria NR (2020). Routes for COVID-19 importation in Brazil. J Travel Med.

[B6] Cascella M, Rajnik M, Cuomo A, Scott C, Dulebohn SC, Napoli RD (2020). Features, Evaluation, and Treatment Coronavirus (COVID-19). StatPearls.

[B7] Chan KH, Peiris JSM, Lam SY, Poon LLM, Yuen KY, Seto WH (2011). The effects of temperature and relative humidity on the viability of the SARS coronavirus. Adv Virol.

[B8] Chin AWH, Chu JTS, Perera MRA Hui KPY, Yen HY, Chan MCW, Peiris M, Poon LLM (2020). Stability of SARS-CoV-2 in different environmental conditions. Lancet Microbe.

[B9] Cui J, Li F, Shi ZL (2019). Origin and evolution of pathogenic coronaviruses. Nat Rev Microbiol.

[B10] de Oliveira SB, Pôrto VBG, Ganem F, Mendes FM, Almiron M, de Oliveira WK, Fantinato FFST, Almeida WAF, Borges APM, Pinheiro HNB (2020). Monitoring social distancing and SARS-CoV-2 transmission in Brazil using cell phone mobility data. Medrxiv.

[B11] Ellwanger JH, Kulmann-Leal B, Kaminski VL, Valverde-Villegas JM, Veiga ABG, Spilki FR, Fearnside PM, Caesar L, Giatti LL, Wallau GL (2020). Beyond diversity loss and climate change: Impacts of Amazon deforestation on infectious diseases and public health. An Acad Bras Cienc.

[B12] Franklin AB, Bevins SN (2020). Spillover of SARS-CoV-2 into novel wild hosts in North America: A conceptual model for perpetuation of the pathogen. Sci Total Environ.

[B13] Góes LGB Ruvalcaba SG, Campos AA Queiroz LH, Carvalho C Jerez JA, Durigon EL, Dávalos LII Dominguez SR (2013). Novel bat coronaviruses, Brazil and Mexico. Emerg Infect Dis.

[B14] Gorbalenya AE, Baker SC, Baric RS, Groot RJ, Drosten C, Gulyaeva AA, Haagmans BL, Lauber C, Leontovich AM, Neuman BW (2020). The species Severe acute respiratory syndrome-related coronavirus: classifying 2019-nCoV and naming it SARS-CoV-2. Nat Microbiol.

[B15] Guan Y, Zheng BJ, He YQ, Liu XL, Zhuang ZX, Cheung CL, Luo SW, Li PH, Zhang LJ, Guan YJ (2003). Isolation and characterization of viruses related to the SARS coronavirus from animals in Southern China. Science.

[B16] Han BA, Kramer AM, Drake JM (2016). Global Patterns of Zoonotic Disease in Mammals. Trends Parasitol.

[B17] Holshue ML, DeBolt C, Lindquist S, Lofy KH, Wiesman J, Bruce H, Spitters C, Ericson K, Wilkerson S, Tural A (2020). First case of 2019 novel coronavirus in the United States. N Engl J Med.

[B18] Jin YH, Cai L, Cheng ZS, Cheng H, Deng T, Fan Y, Fang C, Huang D, Huang L, Huang Q (2020). A rapid advice guideline for the diagnosis and treatment of 2019 novel coronavirus (2019-nCoV) infected pneumonia (standard version). Mil Med Res.

[B19] Kan B, Wang M, Jing H, Xu H, Jiang X, Yan M, Liang W, Zheng H, Wan K, Liu Q (2005). Molecular evolution analysis and geographic investigation of Severe Acute Respiratory Syndrome Coronavirus-like Virus in Palm Civets at an animal market and on farms. J Virol.

[B20] La Rosa G, Bonadonna L, Lucentini L, Kenmoe S, Suffredini E (2020). Coronavirus in water environments: Occurrence, persistence and concentration methods - A scoping review. Water Res.

[B21] La Rosa G, Iaconelli M, Mancini P, Ferraro GB, Veneri C, Bonadonna L, Lucentini L, Suffredini E (2020). First detection of SARS-CoV-2 in untreated wastewaters in Italy. Sci Total Environ.

[B22] Lescure FX, Bouadma L, Nguyen D, Parisey M, Wicky P, Behillil S, Gaymard A, Bouscambert-Duchamp M, Donati F, Hingrat QL (2020). Clinical and virological data of the first cases of COVID-19 in Europe: a case series. Lancet Infect Dis.

[B23] Medema G, Heijnen L, Elsinga G, Italiaander R, Brouwer A (2020). Presence of SARS-Coronavirus-2 RNA in sewage and correlation with reported COVID-19 prevalence in the early stage of the epidemic in the Netherlands. Environ Sci Technol Lett.

[B24] Mellan TA, Hoeltgebaum HH, Mishra S, Whittaker C, Schnekenberg RP, Gandy A, Unwin JT Vollmer MAC, Coupland H Hawryluk I (2020). Report 21: Estimating COVID-19 cases and reproduction number in Brazil. medRxiv.

[B25] Oreshkova N, Molenaar R-J, Vreman S, Harders F, Munnink BBO, Hakze R, Gerhards N, Tolsma P, Bouwstra R, Sikkema R (2020). SARS-CoV2 infection in farmed mink, Netherlands, April 2020. bioRxiv.

[B26] Prata DN, Rodrigues W, Bermejo PH (2020). Temperature significantly changes COVID-19 transmission in (sub)tropical cities of Brazil. Sci Total Environ.

[B27] Rezende LFM, Thome B, Schveitzer MC, Souza-Júnior PRB, Szwarcwald CL (2020). Adults at high-risk of severe coronavirus disease-2019 (Covid-19) in Brazil. Rev Saude Publ.

[B28] Rodriguez-Morales AJ, Gallego V, Escalera-Antezana JP, Méndez CA, Zambrano LI, Franco-Peredes C, Suárez JA, Rodriguez-Enciso HD, Balbin-Ramon GJ, Silvio-Larriera E (2020). COVID-19 in Latin America: The implications of the first confirmed case in Brazil. Travel Med Infect Dis.

[B29] Shi J, Wen Z, Zhong G, Yang H, Wang C, Huang B, Liu R, He X, Shuai L, Sun Z (2020). Susceptibility of ferrets, cats, dogs, and other domesticated animals to SARS-coronavirus 2. Science.

[B30] Souza WM, Buss LF, Candido DS, Carrera J-P, Li S, Zarebski AE, Vincenti-Gonzalez MF, Messina J, Sales FCS, Andrade PS, Prete CA (2020). Epidemiological and clinical characteristics of the early phase of the COVID-19 epidemic in Brazil. medRxiv.

[B31] Spilki FR, da Luz RB, Fabres RB, Soliman MC, Kluge M, Fleck JD, Rodrigues MT, Comerlato J, Cenci A, Cerva C (2013). Detection of human adenovirus, rotavirus and enterovirus in water samples collected on dairy farms from Tenente Portela, Northwest Of Rio Grande do Sul, Brazil. Braz J Microbiol.

[B32] Tu YF, Chien CS, Yarmishyn AA, Lin Y-Y, Luo Y-H, Lin Y-T, Lai W-Y, Yang D-M, Chou S-J, Yang Y-P (2020). A review of SARS-CoV-2 and the ongoing clinical trials. Int J Mol Sci.

[B33] Van Doremalen N, Bushmaker T, Morris DH, Holbrook MG, Gamble A, Williamson BN, Tamin A, Harcourt JL, Thornburg NJ, Gerber SI (2020). Aerosol and surface stability of SARS-CoV-2 as compared with SARS-CoV-1. N Engl J Med.

[B34] Wells CR, Sah P, Moghadas SM, Pandey A, Shoukat A, Wang Y, Wang Z, Meyers LA, Singer BH, Galvani AP (2020). Impact of international travel and border control measures on the global spread of the novel 2019 coronavirus outbreak. Proc Natl Acad Sci U S A.

[B35] Wigginton KR, Ye Y, Ellenberg RM (2015). Emerging investigators series: The source and fate of pandemic viruses in the urban water cycle. Environ Sci Water Res Technol.

[B36] Wilder-Smith A, Freedman DO (2020). Isolation, quarantine, social distancing and community containment: pivotal role for old-style public health measures in the novel coronavirus (2019-nCoV) outbreak. J Travel Med.

[B37] Wong ACP, Li X, Lau SKP, Woo PCY (2019). Global epidemiology of bat coronaviruses. Viruses.

[B38] Wu F, Xiao A, Zhang J, Gu XQ, Lee WL, Kauffman K, Hanage WP, Matus M, Ghaeli N, Endo N (2020). SARS-CoV-2 titers in wastewater are higher than expected from clinically confirmed cases. mSystems.

[B39] Wurtzer S, Marechal V, Mouchel JM, Maday Y, Teyssou R, Richard E, Almayrac JL, Moulin L (2020). Time course quantitative detection of SARS-CoV-2 in Parisian wastewaters correlates with COVID-19 confirmed cases. medRxiv.

[B40] Xiao F, Tang M, Zheng X, Liu Y, Li X, Shan H (2020). Evidence for Gastrointestinal Infection of SARS-CoV-2. Gastroenterology.

[B41] Xu Y, Li X, Zhu B, Liang H, Fang C, Gong Y, Guo Q, Sun X, Zhao D, Shen J (2020). Characteristics of pediatric SARS-CoV-2 infection and potential evidence for persistent fecal viral shedding. Nat Med.

[B42] Zhang YZ, Holmes EC (2020). A Genomic Perspective on the Origin and Emergence of SARS-CoV-2. Cell.

[B43] Zhou P, Lou Yang X, Wang XG, Hu B, Zhang L, Zhang W, Si H, Zhu Y, Li B (2020). A pneumonia outbreak associated with a new coronavirus of probable bat origin. Nature.

